# Human amniotic mesenchymal stromal cells (hAMSCs) as potential vehicles for drug delivery in cancer therapy: an in vitro study

**DOI:** 10.1186/s13287-015-0140-z

**Published:** 2015-08-28

**Authors:** Arianna Bonomi, Antonietta Silini, Elsa Vertua, Patrizia Bonassi Signoroni, Valentina Coccè, Loredana Cavicchini, Francesca Sisto, Giulio Alessandri, Augusto Pessina, Ornella Parolini

**Affiliations:** Department of Biomedical, Surgical and Dental Sciences, University of Milan, Milan, Italy; Centro di Ricerca E. Menni, Fondazione Poliambulanza-Istituto Ospedaliero, Via Bissolati, 57 I-25124, Brescia, Italy; Cellular Neurobiology Laboratory, Department of Cerebrovascular Diseases, Fondazione IRCCS Neurological Institute C. Besta, Milan, Italy

## Abstract

**Introduction:**

In the context of drug delivery, mesenchymal stromal cells (MSCs) from bone marrow and adipose tissue have emerged as interesting candidates due to their homing abilities and capacity to carry toxic loads, while at the same time being highly resistant to the toxic effects. Amongst the many sources of MSCs which have been identified, the human term placenta has attracted particular interest due to its unique, tissue-related characteristics, including its high cell yield and virtually absent expression of human leukocyte antigens and co-stimulatory molecules. Under basal, non-stimulatory conditions, placental MSCs also possess basic characteristics common to MSCs from other sources. These include the ability to secrete factors which promote cell growth and tissue repair, as well as immunomodulatory properties. The aim of this study was to investigate MSCs isolated from the amniotic membrane of human term placenta (hAMSCs) as candidates for drug delivery in vitro.

**Methods:**

We primed hAMSCs from seven different donors with paclitaxel (PTX) and investigated their ability to resist the cytotoxic effects of PTX, to upload the drug, and to release it over time. We then analyzed whether the uptake and release of PTX was sufficient to inhibit proliferation of CFPAC-1, a pancreatic tumor cell line sensitive to PTX.

**Results:**

For the first time, our study shows that hAMSCs are highly resistant to PTX and are not only able to uptake the drug, but also release it over time. Moreover, we show that PTX is released from hAMSCs in a sufficient amount to inhibit tumor cell proliferation, whilst some of the PTX is also retained within the cells.

**Conclusion:**

Taken together, for the first time our results show that placental stem cells can be used as vehicles for the delivery of cytotoxic agents.

## Introduction

In addition to the well-known ability of bone marrow mesenchymal stromal cells (MSCs) to differentiate and exert immunomodulatory effects which make them useful for applications in regenerative medicine, these cells can also migrate to inflammatory microenvironments [1] and tumors [2]. The ability of MSCs to home to sites of injury has brought many researchers to study these cells as vehicles for the delivery of anti-cancer agents to the tumor site. To this end, both gene-modified as well as wild-type MSCs have been used. MSCs have been genetically modified to over-express several anti-tumor factors, such as interleukins, interferons, pro-drugs, oncolytic viruses, anti-angiogenic agents, pro-apoptotic proteins, and growth factor antagonists [3]. Despite promising results in animal models, the genetic manipulation of MSCs for clinical application is not risk-free [4]. We have recently demonstrated that MSCs can behave as chemotherapeutic carriers without genetic manipulation. This was observed for MSCs from bone marrow [5, 6], adipose tissue [7], and dermal fibroblasts [8].

Bone marrow is the best characterized source of adult stem cells; unfortunately, the harvesting procedure is highly invasive and the number, differentiation potential, and maximum life span of MSCs obtained from this tissue significantly decline with the age of the donor [9]. In comparison, placenta is a very attractive MSC source due to its easy, non-invasive, and ethically uncontroversial procurement.

The human amniotic membrane from term placenta has been recently recognized as a valuable source of mesenchymal stromal cells, referred to as hAMSCs [10–12]. hAMSCs have attracted much attention due to their immunomodulatory properties [13], and also due to their paracrine actions and potential applications in regenerative medicine [14]. Interestingly, studies have shown that hAMSCs interact with and modulate the functions of a wide variety of immune cells both in vitro [15–19] and in vivo [20]. Moreover, we have recently demonstrated that hAMSCs can inhibit tumor cell proliferation in vitro [21]. This occurred through cell cycle arrest in the G0/G1 phase, and affected hematopoietic [lymphoid (KG1a, Jurkat), myeloid (KG1, U937)], and non-hematopoietic (Girardi heart, Hela, Saos) tumor cells. Owing to this property and to the ability of amnion-derived stem cells to target tumor sites [22], herein we investigated if hAMSCs were able to uptake the chemotherapeutic agent paclitaxel, and thus be considered as a means of drug delivery for anti-tumor therapy.

## Materials and methods

### Ethics statement

Human term placentae (n = 7) were collected from healthy women after vaginal delivery or caesarean section. Samples were collected after having obtained informed written consent according to the guidelines set by the Ethics Committee for the Institution of Catholic Hospitals (CEIOC). The research project was authorized by Fondazione Poliambulanza.

### Isolation, culture, expansion, and characterization of hAMSC

Human term placentas were processed immediately after birth, as previously described [18]. Briefly, the amnion was manually separated from the chorion and washed extensively in 0.9 % NaCl containing 100U/ml penicillin and 100 μg/ml streptomycin (both from Sigma, St Louis, MO, USA) and 2.5 mg/ml amphotericin B (Sigma (or Sigma-Alrich), St. Louis, MO, USA). Afterwards, the amnion was cut into small pieces (3 × 3 cm^2^). Amnion fragments were sterilized by a brief incubation in 0.9 % NaCl + 2.5 % Eso Jod (Esoform, Italy) and 3 minutes in PBS containing 500U/ml penicillin, 500 μg/ml streptomycin, 12.5 μg/ml amphotericin B and 1.87 mg/ml Cefamezin (Teva Italia Srl, Assago, Italy). Sterilized amnion fragments were then incubated for 9 minutes at 37 °C in HBSS (Sigma (or Sigma-Alrich), St. Louis, MO, USA) containing 2.5 U/ml dispase (VWR International Srl, Milan, Italy). The fragments were digested in complete RPMI 1640 medium (Sigma (or Sigma-Alrich), St. Louis, MO, USA) supplemented with 0.94 mg/ml collagenase (Roche, Mannheim, Germany) and 10 μg/ml DNase (Roche, Mannheim, Germany) for 2.5−3.0 hours at 37 °C. Amnion epithelium fragments were then removed by low-g centrifugation, and mobilized MSC were passed through 100-μm and 70-μm cell strainers and collected by centrifugation. These cells are referred to as human amniotic mesenchymal stromal cells (hAMSCs).

To obtain cells at different passages, freshly isolated P0 hAMSCs were plated at a density of 50 × 10^3^/cm^2^. hAMSCs were cultured at 37 °C and 5 % CO_2_ in DMEM complete medium supplemented with 10 % heat-inactivated fetal bovine serum (FBS, Sigma (or Sigma-Alrich), St. Louis, MO, USA), 2 mM L-glutamine (Sigma (or Sigma-Alrich), St. Louis, MO, USA), 100 U/ml penicillin and 100 μg/ml streptomycin. For phenotype evaluation, hAMSCs were trypsinized and subsequently washed with FACS buffer (0.1 % sodium azide (Sigma-Aldrich) and 0.1 % FBS (Sigma-Aldrich) in PBS). Cells were incubated for 20 minutes at 4 °C with anti-human fluorescein isothiocyanate (FITC), or phycoerythrin- (PE) or allophycocyanin (APC)-conjugated antibodies, or isotype controls (specified below) with 20 mg/ml polyglobin (Gammagard®, Baxter, IL, USA) prepared in PBS with 1 % BSA to block non-specific binding. After incubation cells were washed with FACS buffer. Dead cells were gated out by propidium iodide (PI) staining (for cell surface staining). The clones and suppliers of the monoclonal antibodies used are as follows: CD44 (clone L178), CD73 (AD2), CD90 (5E10), CD45 (2D1), HLA-DR (TU36), CD105 (266), CD13 (L138), and HLA-ABC (G46-2.6) were all purchased from BD Bioscience, San Jose, CA, USA.

Intracellular P-glycoprotein (P-gp) expression was analyzed using a mouse anti-human monoclonal antibody (clone JSB-1, Chemicon International Merck Millipore, Billerica, MA, USA). Briefly, cells were fixed and permeabilized using BD CytoFix/CytoPerm (BD Biosciences, San Jose, CA, USA) for 20 minutes at 4 °C, washed twice with Perm/Wash Buffer 1X (BD Biosciences, San Jose, CA, USA), and incubated with P-gp antibody for 25 minutes at room temperature. After two washes in Perm/Wash Buffer 1X, cells were incubated with goat anti-mouse polyclonal immunoglobulins/RPE Goat F(ab’)2 (DAKO Corporation, Denmark), and washed prior to acquisition. Cells were acquired on a FACS Calibur machine using CellQuest Software (BD Biosciences, San Jose, CA, USA) and results were analyzed using FCS Express 4 (De Novo Software, Los Angeles, CA, USA). IgG1 (clone X40, BD Biosciences, San Jose, CA, USA) and IgG2b (clone MG2b-57, Biolegend, San Diego, CA, USA) were used as isotype controls. Quantification of P-gp expression was performed by determining the mean fluorescence intensity (MFI) ratio as follows: MFI of P-gp/MFI isotype control.

### Sensitivity of hAMSCs to Paclitaxel

Paclitaxel (PTX) was purchased from AdipoGen (Vinci-Biochem, Vinci, Italy), diluted in dimethylsulfoxide to a concentration of 5 mg/ml, and stored at −20 °C in 5-μl aliquots. PTX was thawed immediately prior to use and diluted in culture medium to obtain the desired concentration.

The anti-proliferative and cytotoxic effects of PTX on hAMSCs were evaluated in 96-multiwell plates (Corning, Corning, NY, USA) by first seeding 2,000 and 10,000 cells/well, respectively, in 100 μl/well of complete medium. The cells were then incubated for 24 hours (cytotoxicity test) or 7 days (anti-proliferative assay) with 10-fold dilutions of PTX (from 1 ng/ml to 10,000 ng/ml). At the end of the incubation, cell proliferation and viability were evaluated by a 3-(4,5-dimethylthiazol-2-yl)-2,5-diphenyltetrazolium bromide (MTT) assay, as previously described [5]. The inhibitory concentrations (IC_50_ and IC_90_) were determined according to the Reed and Muench formula [6] or by linear regression analysis.

### Paclitaxel priming of hAMSCs

Sub-confluent cultures (3−4 × 10^5^ cells) of hAMSCs were exposed to 2,000 ng/ml of PTX. Twenty-four hours later, the cells were collected, counted, and seeded at the concentration 10^5^ cells/ml, according to a previously described protocol [5]. Conditioned media from primed hAMSCs (hAMSCsPTX-CM) were collected after 48 hours, centrifuged at 2,500 g for 15 minutes to discard cell debris, aliquoted, and stored at −70 °C. The remaining cells were trypsinized and then lysed by resuspension (10^6^ cells/ml) in bi-distilled water and four freeze/thaw cycles. After centrifugation at 2,500 g for 15 minutes, cell debris was discarded and the lysates (hAMSCsPTX-LYS) were aliquoted and stored at −70 °C.

In order to evaluate the release of PTX over time, hAMSCsPTX-CM were collected at different timepoints (48, 72, 120 hours), and after each collection hAMSCsPTX-CM was replaced with fresh medium. Both conditioned media (CM) and lysates (LYS) were tested *in vitro* for their anti-proliferative activity against CFPAC-1, a human ductal pancreatic adenocarcinoma cell line highly sensitive to PTX. The values obtained were normalized by CM and LYS from untreated hAMSCs.

### Modulation of hAMSC sensitivity to PTX with verapamil

Verapamil (VP), a P-gp inhibitor, was purchased as a 5.5 mM solution for i.v. injection (Isoptin, Abbott, Rome Italy). The modulation of PTX sensitivity was evaluated through a proliferation assay as reported above. Cells were seeded in the presence of increasing PTX concentrations and 20 μM of VP, a dose previously demonstrated to affect PTX sensitivity in a murine bone marrow stromal cell line [5].

### *In vitro* anti-proliferative assay on CFPAC-1 of PTX, CM and LYS from PTX-primed hAMSCs

The effects of PTX, hAMSCsPTX-CM, and hAMSCsPTX-LYS were studied on CFPAC-1 using a MTT assay. Briefly, two-fold serial dilutions of pure PTX, PTX-CM, or PTX-LYS were prepared in 100 μl of culture medium/well in 96-multiwell plates (Corning, USA) and then 1,000 tumor cells were added to each well. Tumor cell viability was evaluated by the MTT assay after 7 days of incubation at 37 °C and 5 % CO_2_. The percentages of viability were calculated by dividing the optical density of tumor cells grown in PTX-CM or PTX-LYS by the optical density found in cells grown in the same dilution of CM or LYS obtained from control hAMSCs. The anti-tumor activity of PTX-CM and PTX-LYS were compared to that of pure PTX and expressed as paclitaxel equivalent concentration (PEC_CM_ and PEC_LYS_, respectively) according to the following algorithm:

$$ \mathrm{P}\mathrm{E}\mathrm{C}\ \left(\mathrm{ng}/\mathrm{ml}\right) = \left({\mathrm{IC}}_{50}\mathrm{P}\mathrm{T}\mathrm{X}/{\mathrm{V}}_{50}\right) \times 100. $$

V_50_ is the volume (μl/well) of CM or LYS able to inhibit CFPAC-1 proliferation by 50 %; IC_50_PTX is the concentration (ng/ml) of pure paclitaxel able to inhibit CFPAC-1 proliferation by 50 %. The amount of PTX internalized and released by a single hAMSC cell (PEC_CM,_ picograms (pg)/cell) and the amount of PTX internalized and retained inside each hAMSC (PEC_LYS,_ pg/cell) was determined as follows:

$$ \mathrm{P}\mathrm{E}\mathrm{C}\left(\mathrm{pg}/\mathrm{cell}\right) = \mathrm{P}\mathrm{E}\mathrm{C}\left(\mathrm{ng}/\mathrm{ml}\right) \times \mathrm{C}\mathrm{M}\ \mathrm{or}\ \mathrm{L}\mathrm{Y}\mathrm{S}\ \mathrm{volume}\left(\mathrm{ml}\right)\times 1000/\mathrm{number}\ \mathrm{of}\ \mathrm{cells}\ \mathrm{seeded}. $$

The sum PEC_CM+_ PEC_LYS_, both expressed as pg/cell, indicates the total amount of PTX incorporated by a single cell after 24 hours of exposure to 2,000 ng /ml of PTX.

### Statistical analysis

Comparison between different hAMSC donors was performed by a multiple comparison post-test (one-way analysis of variance (ANOVA)) and *p* values <0.05 were considered significant. Values represent mean ± standard deviation (SD).

## Results

### hAMSC characterization

**Fig. 1:**
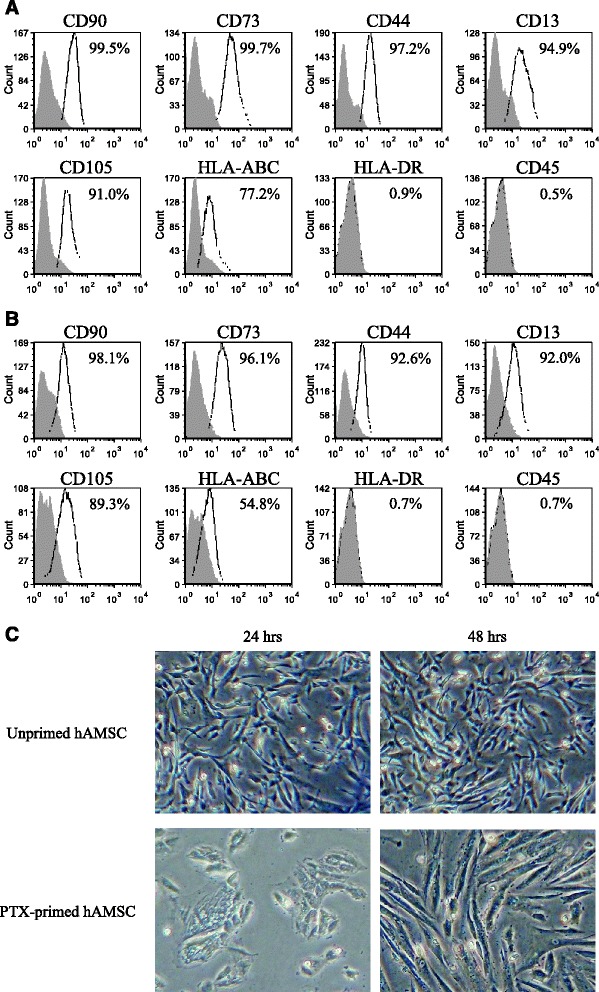
Characterization of human amniotic mesenchymal stromal cells (*hAMSCs*). Phenotype of unprimed (**a**) and paclitaxel (*PTX*)-primed (**b**) hAMSCs. The percentage of positive cells is indicated in each plot. Cell morphology is shown in panel **c**, magnification × 4. The images on the *left* show unprimed hAMSC (*top*) or hAMSC after the 24-hour treatment with 2,000 ng/ml of PTX (*bottom*). The images on the *right* show unprimed hAMSC (*top*) or hAMSC at the time at which conditioned media and lysates were collected and tested for their anti-proliferative activity against CFPAC-1 (*bottom*, 48 hrs)

hAMSCs were used at either passages 3 or 4 and were analyzed for phenotype and morphology. Figure 1 reports the phenotype of hAMSCs, which is in line with previously published studies showing the expression of mesenchymal lineage markers CD90, CD73, CD44, CD13, HLA-ABC, and CD105, and absence of CD45 and HLA-DR [15, 23]. Interestingly, we did not notice any substantial differences in marker expression between unprimed (Fig. 1a) and PTX-primed (Fig. 1b) hAMSCs.

The morphology of unprimed and PTX-primed hAMSCs is shown in Fig. 1c. Twenty-four hours after the addition of PTX, hAMSCs were more enlarged and had increased granularity when compared to their unprimed controls. After 48 hours, PTX-primed cells recovered their fibroblast-like morphology, similar to their unprimed counterparts.

### hAMSC sensitivity to PTX

**Fig. 2:**
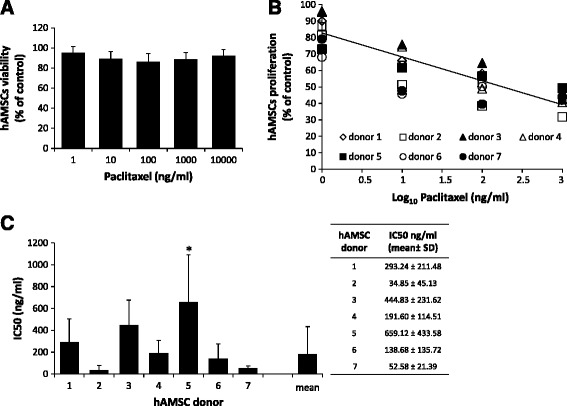
Paclitaxel (PTX) sensitivity of human amniotic mesenchymal stromal cells (*hAMSCs*). **a** Twenty-four-hour cytotoxicity assay of hAMSCs in the presence of PTX. *Bars* represent mean value ± SD for five donors. **b** Seven-day anti-proliferation assay of hAMSCs (seven donors) in the presence of 10-fold serial dilutions of PTX (linear regression analysis). **c** Paclitaxel half maximal inhibitory concentration (*IC*_*50*_) values (expressed as ng/ml) assessed by linear regression analysis in the anti-proliferation assay. For each donor, the mean value ± SD of at least two independent experiments is reported. The analysis of IC_50_ values by a multiple comparison test showed that only the IC_50_ of donor 5 is significantly different from mean value (*)

hAMSCs were highly resistant to PTX cytotoxicity when evaluated 24 hours after treatment. Their viability was >90 % even at the highest PTX concentration tested (10 μg/ml), (Fig. 2a). Based on these results, we established that a PTX treatment time of 24 hours was suitable, and that a 2,000 ng/ml dose would be used for experiments with hAMSCs. This is in accordance with a previous study that used the same concentration and treatment duration for priming bone marrow (BM)-MSCs with PTX [5].

Concerning the effects of PTX on the proliferation of hAMSCs (Fig. 2b and c), we observed significant heterogeneity in PTX sensitivity amongst hAMSCs from the seven donors: the IC_50_ values ranged from 34.85 ng/ml to 659.12 ng/ml (Fig. 2c).

### Evaluation of PTX release from primed hAMSCs

**Fig. 3:**
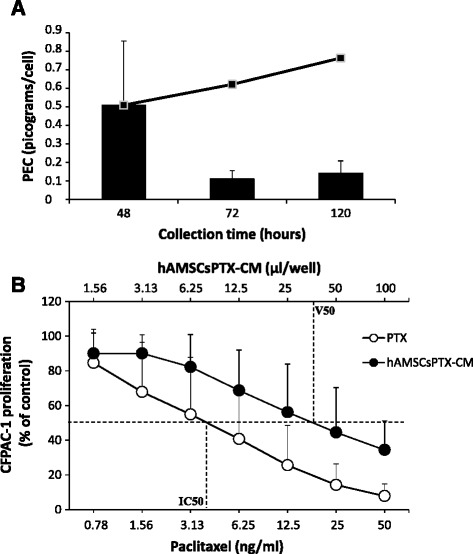
Paclitaxel (*PTX*) uptake/release by human amniotic mesenchymal stromal cells (*hAMSCs*). **a** Release of PTX over time evaluated in four out of seven donors. *Bars* represent the amount of PTX (expressed as picograms/cell) released at each time point, and the curve expresses the total amount of PTX released over time. **b** Proliferation curves of CFPAC-1 in the presence of serial dilutions of PTX (*white circles*) or conditioned media from PTX-primed hAMSCs (*hAMSCsPTX-CM*) (*black circles*). Each point represents the mean value ± SD. The curve of hAMSCsPTX-CM represents mean values obtained from seven donors. The half maximal inhibitory concentration (*IC*_*50*_) and the volume of CM able to inhibit tumor growth by 50 % (*V*_*50*_) are shown. *PEC* paclitaxel equivalent concentration

After having observed that hAMSCs were resistant to the cytotoxic effect of PTX, we then sought to investigate if they could take up and release the drug in culture, a characteristic previously observed using MSC from other sources [5]. To this end, we evaluated the release of the drug over time by collecting and replacing culture medium at different time intervals. hAMSCsPTX-CM was collected at 48, 72, and 120 hours after priming with PTX (Fig. 3a). For the four out of seven donors tested, we observed that the release of the drug was highest after 48 hours, and then decreased over time. PTX was detected in the CM collected from hAMSCs cultured for up to 120 hours after priming. In order to investigate if the PTX released from hAMSCs was sufficient to inhibit tumor cell proliferation, hAMSCs were subcultured for an additional 48 hours after priming, which represented the time point at which we observed the highest release of the drug and is also in accordance with a previously described protocol [5]. The anti-proliferative potential of hAMSCsPTX-CM was evaluated on CFPAC-1, a human pancreatic adenocarcinoma cell line highly sensitive to PTX (IC_50_ = 3.97 ± 4.48 ng/ml, n = 47), and compared to pure PTX (Fig. 3b). The hAMSCsPTX-CM from all seven donors produced a dose-dependent, anti-proliferative effect on CFPAC-1 (Fig. 3b).

**Fig. 4:**
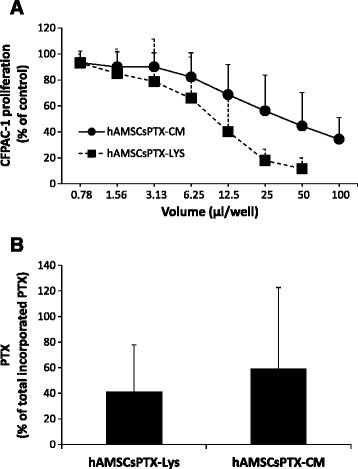
Evaluation of paclitaxel (*PTX*) internalized by human amniotic mesenchymal stromal cells (hAMSCs) but not released into culture medium. **a** Proliferation curves of CFPAC-1 in the presence of serial dilutions of conditioned media from PTX-primed hAMSCs (*hAMSCsPTX-CM*) (*solid line*) or lysates from PTX-primed hAMSCs (*hAMSCsPTX-LYS*) (*dashed line*). Five different hAMSC donors were tested. **b** The graph shows the amount of PTX incorporated and released by hAMSCs (CM) and the amount incorporated and retained inside the cells (LYS), expressed as percentages of the total incorporated PTX, considered 100 %. *Bars* represent the mean values ± SD. Five different hAMSC donors were tested. The difference between CM and LYS was not statistically significant (*p* >0.05)

The highest release of PTX was observed after 48 hours and represented approximately one half (59.3 %) of the incorporated drug, which was comparable to a release of approximately 0.51 ± 0.29 pg/cell (Fig. 3a). This suggests that some PTX was retained by the cells and not released, an observation previously also reported for MSCs from bone marrow [5]. To evaluate the amount of PTX internalized but not released into hAMSCsPTX-CM, at the end of the 48 hours of subculture and after the collection of hAMSCsPTX-CM (release phase), cells were trypsinized and lysed. The presence of PTX in the lysates (hAMSCsPTX-LYS) was then tested by analyzing the effects of hAMSCsPTX-LYS on the proliferation of CFPAC-1 tumor cells. Figure 4a shows that hAMSCsPTX-LYS was able to inhibit CFPAC-1 proliferation with a trend similar to hAMSCsPTX-CM, suggesting that a proportion of the internalized PTX is not released into the culture medium, but rather, is retained inside primed cells. The difference observed between hAMSCsPTX-LYS and hAMSCsPTX-CM in inhibiting CFPAC-1 tumor cell proliferation was not significant, and could be influenced by the number of hAMSCs and the volume of diluent used for the preparations of CM and LYS. By considering the PEC values found in hAMSCsPTX-CM and hAMSCsPTX-LYS, we calculated the percentages of PTX released and retained by the cells, respectively. As shown in Fig. 4b, more than 50 % of the incorporated PTX was released by the primed cells during the subculture phase (59.02 ± 63.56 %, mean value obtained with five out seven donors tested) and the remaining amount (40.98 ± 36.81 %) was retained inside hAMSCs primed with PTX.

### Effect of verapamil on PTX sensitivity and uptake/release ability of hAMSCs

**Fig. 5:**
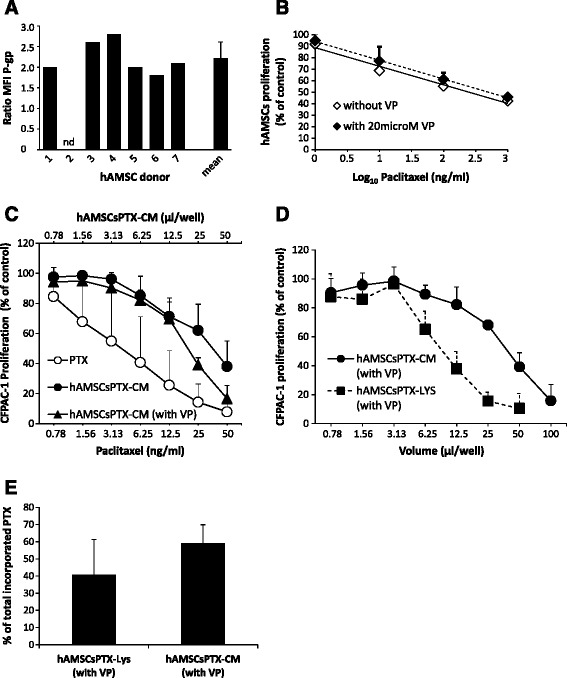
Effect of verapamil on paclitaxel (*PTX*) toxicity and PTX uptake/release by human amniotic mesenchymal stromal cells (*hAMSCs*). **a** P-glycoprotein (*P-gp*) expression is represented as the ratio of mean fluorescence intensity (*MFI*) for each donor: *nd* not determined. **b** Proliferation of hAMSCs in the presence of PTX and 20 μM verapamil (*VP*). Half maximal inhibitory concentration (IC_50_) values (mean ± SD) were calculated by linear regression analysis. **c** Proliferation curves of CFPAC-1 in the presence of serial dilutions of PTX (*white circles*), conditioned media from PTX-primed hAMSCs (*hAMSCsPTX-CM*) (*black circles*) or hAMSCsPTX-CM collected from cells primed with PTX in the presence of 20 μM VP (*black triangles*). **d** Proliferation curves of CFPAC-1 in the presence of serial dilutions of hAMSCsPTX-CM (solid line) or hAMSCsPTX-LYS (dashed line) from PTX primed hAMSCs. Both CM and LYS were obtained from hAMSCs primed with PTX in the presence of 20 μM VP. **e** Amount of PTX incorporated and released by hAMSCs (CM) and the amount incorporated and retained inside the cells (LYS), expressed as percentages of the total incorporated PTX, considered 100 %. hAMSCs were primed in the presence of 20 μM VP. Bars represent the mean values ± SD. The difference between CM and LYS was not statistically significant (*p* >0.05). To evaluate the effect of VP, hAMSCs from two donors were used

P-gp has been described to be associated with drug resistance through an increased drug efflux from tumor cells [24]. In order to investigate whether P-gp underlies the mechanism by which hAMSC are resistant to PTX, we first analyzed P-gp expression in hAMSCs from the seven donors. P-gp was expressed in six out of seven hAMSC donors analyzed, with a mean ratio of fluorescence intensity (MFI) of 2.2 ± 0.39 (Fig. 5a). Next, we investigated if blocking the pump with verapamil (VP), an inhibitor of P-gp, could alter the sensitivity of hAMSCs to PTX. As shown in Fig. 5b, the presence of VP had no effect on hAMSC sensitivity to the anti-proliferative activity of PTX. Furthermore, as shown in Fig. 5c and d, the presence of 20 μM VP during the PTX uptake phase did not significantly alter the amount of drug internalized and subsequently released by hAMSCs. In fact, in line with previous observations (Fig. 4b), 59.19 % of the incorporated PTX was released into the culture medium and 40.81 % was retained inside the cells (Fig. 5e).

## Discussion

For the first time we demonstrate that MSCs from the amniotic membrane of human term placenta can be loaded with PTX and can release the drug over time. Notably, the drug released from hAMSCs is able to inhibit tumor cell proliferation *in vitro*. The findings described herein make these cells interesting candidates for drug delivery vehicles, also considering that they are able to inhibit tumor cell proliferation per se under specific culture conditions *in vitro* [21].

We show that hAMSCs are resistant to the cytotoxic effect of PTX, a drug known for its strong anti-tumor [25] and anti-angiogenic activities [26], and currently used to treat advanced solid tumors [27–30]. Resistance to PTX has been reported in MSC from other sources (BM [5], adipose tissue [7] and dermal fibroblasts [8]).

We observed significant heterogeneity in the ability of PTX to inhibit proliferation of hAMSCs from seven healthy donors; indeed, the range of IC_50_ values was 34.85−659.12 ng/ml. Interesting, placental MSCs from all seven donors had higher resistance to PTX when compared to MSCs from other sources. In our previous studies, MSCs from alternative sources had more homogeneous PTX sensitivity, regardless of the donors. IC_50_ values for BM-MSCs, AT-MSCs, and dermal fibroblasts were 4.07 ± 1.75 ng/ml [5], 2.55 ± 1.02 ng/ml [7], and 7.01 ± 2.17 ng/ml [8], respectively.

Even though most of the incorporated drug was released within 48 hours, it is interesting to note that drug was released into the culture medium for up to 120 hours after priming. Although the mechanism of PTX binding to microtubules has been extensively studied [25], very little is known about the molecular mechanisms at the basis of the drug resistance of MSCs, or the capacity of these cells to accumulate and release PTX. In previous experiments, we demonstrated the expression of P-gp, the first discovered and the best-characterized of drug-efflux transporters, by human BM-MSCs [5]. Over the last few years, several studies have been performed to better understand the role that placenta plays in distributing pharmacological agents within the maternal and fetal compartments [31] and the presence of several drug efflux proteins in placenta has been demonstrated [32]. For example, the expression of the breast cancer resistant protein (BCRP) has been previously described [33], while other authors confirmed the presence of P-gp in syncytiotrophoblast cells [34, 35]. It is interesting to note that, despite its presence, P-gp protein does not seem to have a role in PTX transport in human placenta [36]. The lack of correlation between P-gp expression and PTX transport could be explained by the inverse relationship between protein expression and activity, and by the polymorphism of the *MDR1* gene [37]. Furthermore, placenta is known to express a spectrum of metabolizing enzymes [37]; among them are drug-metabolizing CYP enzymes (such as CYP1A and CYP2E1). Further studies are therefore warranted to better clarify other mechanisms of resistance, which could also be acting in placental MSCs, such as mutations in the tubulin gene [38], presence of different tubulin isotypes [39], or altered dynamics of microtubules [40]. Studies to verify the possible role of survivin, which has been shown to regulate cell division and/or survival in the presence of Taxol [41], would also provide relevant insight.

Notwithstanding the mechanism by which hAMSCs take up and secrete PTX, our data demonstrate for the first time that through a simple process of *in vitro* priming, these cells incorporate PTX in an amount sufficient to inhibit tumor cell proliferation *in vitro*.

Amongst the different MSC sources investigated and identified over the years, the human term placenta has drawn increased interest mainly due to its non-invasive procurement and large cell yield. Placental MSCs also share basic properties with MSCs from other sources, such as bone marrow. In addition, they offer significant advantages for application in the clinic due to their immunomodulatory capacities [15, 19, 20], making them very attractive for transplantation in allogeneic settings. Therefore, in addition to the advantages of using placenta as a source of MSCs, their ability to take up and release PTX over time in a sufficient amount to inhibit tumor cell proliferation could surely have a significant impact in the context of targeted cancer therapy.

## Conclusions

Herein, we demonstrate that mesenchymal stromal cells from the amniotic membrane of human term placenta (hAMSCs) are highly resistant to the cytotoxicity of PTX. Of note, hAMSCs are able to take up, retain, and release PTX, as shown by the anti-proliferative effects exerted by lysates and conditioned medium obtained from PTX-primed hAMSCs on tumor cells *in vitro*. Interestingly, we also show that P-gp, even though expressed by hAMSCs, does not seem to be implicated in hAMSC resistance to PTX, as shown by the fact that blocking P-gp with verapamil had no effect on hAMSC sensitivity to the anti-proliferative activity of PTX. Furthermore, P-gp inhibition did not significantly alter the amount of drug internalized and subsequently released by hAMSCs.

Taken together, our results show that placental stem cells can be used as vehicles for delivery of cytotoxic agents, thus putting forth a new potential strategy for the delivery of cytotoxic loads to tumors, and at the same time contributing to our understanding of placental MSC, a rapidly evolving field of interest.

## Abbreviations

ANOVA: analysis of variance
APC: allophycocyanin
BM: bone marrow
BCRP: breast cancer resistant protein
BSA: bovine serum albumin
CYP: cytochrome
DMEM: Dulbecco's modified Eagle medium
FACS: fluorescence automated cell sorting
FBS: fetal bovine serum
FITC: fluorescein isothiocyanate
hAMSC: human amniotic mesenchymal stromal cells
hAMSCsPTX-CM: conditioned media from paclitaxel-primed hAMSCs
hAMSCsPTX-LYS: lysates from paclitaxel-primed hAMSCs
HBSS: Hank's Balanced Salt Solution
IC_50_: half maximal inhibitory concentration
MDR1: multi-drug resistance
MFI: mean fluorescence intensity
MSC: mesenchymal stromal cells
MTT: (3-(4,5-dimethylthiazol-2-yl)-2,5-diphenyltetrazolium bromide)
PBS: phosphate-buffered saline
PE: phycoerythrin
PEC: paclitaxel equivalent concentration
P-gp: P-glycoprotein
PI: propidium iodide
PTX: paclitaxel
RPMI: Roswell Park Memorial Institute medium
V_50_: volume of conditioned media or lysates able to inhibit tumor growth by 50 %
